# Chromosomal Aberrations in Blood Lymphocytes as Predictors of Respiratory Function After Stereotactic Lung Irradiation

**DOI:** 10.3389/fonc.2021.829972

**Published:** 2022-01-27

**Authors:** Zsuzsa S. Kocsis, Gyöngyi Farkas, András Bajcsay, Márta Kun-Gazda, József Lövey, Gyula Ostoros, Tamás Pócza, András Herein, Katalin Ladányi, Gábor Székely, Zsolt Markóczy, Zoltán Takácsi-Nagy, Csaba Polgár, Zsolt Juranyi

**Affiliations:** ^1^ Department of Radiobiology and Diagnostic Onco-Cytogenetics, Centre of Radiotherapy, National Institute of Oncology, Budapest, Hungary; ^2^ Centre of Radiotherapy, National Institute of Oncology, Budapest, Hungary; ^3^ Semmelweis University, Faculty of Medicine, Department of Oncology, Budapest, Hungary; ^4^ National Koranyi Institute of Pulmonology, Budapest, Hungary

**Keywords:** SABRT, radiotherapy, lung cancer, pulmonary function tests, chromosomal aberrations, radiosensitivity, SBRT

## Abstract

Due to the profound difference in radiosensitivity of patients and various side effects caused by this phenomenon, a radiosensitivity marker is needed. Prediction by a marker may help personalise the treatment. In this study, we tested chromosomal aberrations (CA) of *in vitro* irradiated blood as predictor of pulmonary function decrease of nonsmall cell lung cancer (NSCLC) patients and also compared it with the CAs in the blood of irradiated patients. Peripheral blood samples were taken from 45 lung cancer patients before stereotactic radiotherapy (SBRT) and immediately after the last fraction and 3, 6, 9, 12, 15, 18, 21, and 24 months later. Respiratory function measurements were performed at the same time. Diffusing capacity of lung for carbon monoxide (DLCO), forced vital capacity (FVC), forced expiratory volume in 1 s (FEV1s), and FEV1s/FVC (FEV1%) were monitored. Metaphase preparations of lymphocytes were made with standard procedures, and chromosome aberrations were analysed. In our cohort, the 36-month local relapse-free survival was 97.4%, and the distant metastasis-free survival was 71.5% at 36 months. There was no change in the mean of the pulmonary function tests (PFTs) after the therapy. However, there was a considerable variability between the patients. Therefore, we subtracted the baseline and normalised the PFT values. There were significant decreases at 12–24 months in relative FEV1s and relative FEV1%. The tendentious decrease of the PFTs could be predicted by the *in vitro* chromosome aberration data. We also found connections between the *in vitro* and *in vivo* CA values (i.e., dicentrics plus rings after 3 Gy irradiation predicts dicentric-plus-ring value directly after the radiotherapy/V_54 Gy_ (*p* = 0.001 24.2%)).

We found that—after further validation—chromosome aberrations resulted from *in vitro* irradiation before radiotherapy can be a predictive marker of pulmonary function decrease after lung irradiation.

## Introduction

Radiotherapy is a crucial modality for treating lung cancer, which still leads the morbidity and mortality statistics worldwide. Although surgical resection is the standard of care for stages I–II nonsmall cell lung cancer (NSCLC) (1, 2), a high percentage of these patients are inoperable due to comorbidities, mostly chronic obstructive pulmonary disease (COPD) (3–5). Therefore, stereotactic body radiotherapy (SBRT) has an emerging role in the treatment of patients with medically inoperable tumours. Compared with standard radiation, stereotactic body radiation therapy (SBRT) offers larger fractions, smaller field, and shorter, therefore, more favourable overall treatment time (6, 7).

On the other hand, there may be large volumes irradiated with low dose (especially at arc radiation techniques) compared with standard radiotherapy which raised questions of possible pulmonary function decrease. Accordingly, SBRT seems to be well tolerated at population level in the medically inoperable patient group, but the diffusing capacity of the lung carbon monoxide (DLCO) or forced vital capacity (FVC) results are controversial (8–10). The cause might be that the average lung function values after SBRT of the whole patient group may not change, but the individuals may exhibit significant increases or decreases. Stephens et al., for example, found that 10% of the patients experienced at least 14.8% decline in FEV1%, while another 10% experienced an increase of at least 12.7% (11). They also showed similar DLCO variability. The extreme differences and possible unexpected severe radiogenic toxicities can be caused by the individual radiosensitivity just as in the case of every radiotherapy (12). To judge the risk of pulmonary fibrosis, the baseline clinical and treatment planning data, such as tumour volume, stage, and isodose volumes may not be enough (13).

Therefore, the main objective of predictive radiosensitivity testing is to screen candidates in order to pinpoint patients at risk (14–18). The relationship between radiation therapy-related side effects and frequencies of chromosomal aberrations (CAs) has already been correlated to individual radiosensitivity in former publications; however, there was no consensus in the results (19–22). The method is very cheap and robust; therefore, it can be a considerable choice beside genetic tests. It was suggested that this correlation can be later used in the clinical practice (23). Micronucleus method is a highly similar method and is also suggested as a predictive biomarker in clinical studies; however, its results are similarly contradictory (24–28).

In our study, we compared chromosomal aberrations *in vitro* and *ex vivo* before and after irradiation treatments and follow-up in patients. We tested whether the chromosomal radiosensitivity of *in vitro*-irradiated lymphocytes (3 and 6 Gy) could be used to predict the risk of decrease in pulmonary functions (FVC, DLCO) after radiotherapy of lung cancer patients.

## Methods

### Patients

This study was approved by the Hungarian Ethical Committee, ETT TUKEB (*23546-3/2017/EKU*) and was conducted following the principles of the Helsinki Declaration. All subjects were informed about the aim of the study and gave their written consent to participate.

We enrolled 45 patients between January 2016 and February 2019. Tumours with a diameter below 3 cm were treated, but the TNM status was diverse: 68.9% of the patients had T1 tumour and 15.6% T2 tumour; the others had T4 (4.4%) or tumour not staged. We also treated two metastases, which were the disseminations of a gastrointestinal and a renal primary tumour. These were excluded from survival analysis but included in CA and pulmonary function test (PFT) evaluation.

### Treatment Procedure

Patients were treated with stereotactic body radiation therapy based on the 4D-CT with 3 mm slice thickness taken in free breath mode. Critical structures, including spinal cord, heart, brachial plexus, and oesophagus were outlined, and dose constraints are used according to accepted standards (29) (**Supplementary Table S1**). Combining the gross target volume (GTVs) (determined by contouring the macroscopic tumour using lung window) in seven respiratory positions without extension, internal target volume (ITV) was generated. Planning target volume (PTV) was produced by the 0.6-cm extension of the ITV. An eighth, average CT reconstruction was also generated and used for dose calculation and optimisation. Forty-two patients were treated with 8 × 7.5 Gy, and three patients were treated with 5 × 12 Gy. We used volumetric modulated arc therapy (VMAT) technique, 6 MV accelerator energy with flattening filter free (FFF) mode and 1,400 MU/min dose rate. We used Varian Eclipse 13.7 planning system (AAA calculation algorithm) and TrueBeam linear accelerator.

### Lymphocyte Culture and Chromosome Aberrations

Before radiotherapy, immediately after the irradiation, and later every 3 months, 10 ml of blood was drawn from the patients. The 3- or 6-Gy *in vitro* irradiation of blood taken before the therapy was performed under the same conditions (1,400 MU/min, 6 FFF) as the radiotherapy. The irradiation was executed in a water phantom in cryotubes positioned in the isocenter (the bottom of the cryotube was 4 cm below the surface of the water). The lymphocyte cultures were induced to proliferate with phytohaemagglutinin M (2% *v*/*v*, Gibco, Waltham, MA, USA). Both the *in vitro*-irradiated and nonirradiated blood samples were cultured by standard cytogenetic techniques in cell culture medium (RPMI-1640, 15% bovine serum albumin; 100 U/ml penicillin, 100 µg/ml streptomycin) for 48 h. For arresting cells in metaphase, the cultures were incubated with 0.1 μg/ml Colcemid (Gibco) for two more hours. Cell cultures were treated with hypotonic solution (0.075 M KCl) for 15 minutes at 37°C; the fixation step was repeated with cold methanol-acetic acid five times (3:1). The cell suspension was dropped on glass slides, dried, and stained with 3% Giemsa dye.

### Analysis of Chromosomal Aberrations

At least 100 metaphases/sample were analysed with 1,500× magnification by two experienced analysers and customary given as aberration/100 cells. The following aberrations were evaluated: chromatid breaks, exchanges, chromosome fragments, dicentrics, rings, and translocations (which was distinguishable with Giemsa staining). One dicentric or ring (centric and acentric) and their associated fragment were counted together as one aberration. Excess terminal and interstitial deletions are summed up and analysed as “chromosome fragments”. The total aberration count is the sum of these values, while aberrant cell value is the number of cells with any aberration. We applied the requirements of the ICPEMC (30) guideline.

### Pulmonary Function Tests

We analysed DLCO and FVC before radiotherapy, directly after the last fraction and 3, 6, 9, 12, 15, 18, 24, and 36 months after the therapy within 1 week after the blood draw. We calculated relative changes by subtracting baseline value from the actual data and dividing the difference with the baseline value.

### Statistical Analysis

The chromosomal aberration values and yields of aberrant cells were given per 100 cells, as customary. As the distribution of aberration values differ from the normal distribution, Mann-Whitney test was performed to analyse differences between CAs. We performed univariate regression analysis to compare CA values of in vitro-irradiated blood and PFT values. This method was also applied to connect in vitro and in vivo CA values. Outliers were detected by ROUT method in GraphPad Prism 8 (San Diego, CA, USA) (31). We did not consider data series with less than fifteen cases. (The first patient was excluded from in vitro CA analysis as the irradiation was performed under slightly different conditions as the irradiation of the other blood samples.) The p-value of <0.05 was considered significant in every test. Survival functions were analysed using Kaplan-Meier curves. For data analysis and presentation, Minitab 18.1, OriginPro 8.5, and IBM SPSS statistics 25.0 software packages were used.

## Results

### Epidemiology

We recruited 45 patients (**Table 1**) between 52 and 84 years of age. There was a female predominance. Most patients (92.8%) had a history of smoking, and COPD was a comorbidity in 77.8%. Median DLCO was 48.8%, and the mean FVC was 84.9%.

**Table 1:** Patient characteristics.

Characteristic	
**Median age (range)**	69 years (52–84)
**Gender: female/male**	25/20 (55.6% vs. 44.4%)
**Stage**	
T1, T2	32/9
T4^a^, T_X_	2/2
**Histology**	
Planocell	3
Adenocarcinoma	9
Other, poorly diff.	1
No biopsy, PET/CT	32
**Smoking status (no information for 3 patients)**	
History of smoking: never	3 (7.1%)
Active smoking/exsmoker	10/29 (92.8%)
**Pulmonary function (mean ± SD)**	
FVC %	84.9 ± 19.8
DLCO (as % of predicted)	48.8 ± 21.9
FEV1 (%)	68.4 ± 25.3
FEV1s (L)	1.6 ± 0.6
**COPD/non-COPD**	35/10
**Treatment**	
8 × 7.5 Gy	42
5 × 12 Gy	3

a Multifocal tumour or second tumour on the contralateral side.

### Treatment Efficacy, Survival Curves

For all patients, the local relapse-free survival was 97.4% at 36 months, and the regional relapse-free survival was similarly favourable (93.6%) (**Supplementary Figures S1A, B**). On the other hand, we observed 71.5% distant metastasis-free survival (median was 40 months (35.9–44.1 95% CI)), which characterises the disease more than it describes the effectivity of the radiotherapy (**Supplementary Figure S1C**). However, 84.2% of the patients did not need any other treatment for 3 years (**Supplementary Figure S1D**). The 3-year lung cancer-specific survival was 83.6%, and due to the comorbidities, the overall survival was 68.9% (**Supplementary Figures S1E, F**).

### Chromosomal Aberrations *In Vitro*

In order to assess the predictive power of the chromosome aberration method, we irradiated the blood samples taken before the therapy with 3 and 6 Gy under the same conditions as patients were treated (dose rate, photon energy) (**Supplementary Table S2**). (One part of the sample served as unirradiated control to detect possible chromosomal aberrations present before the beginning of the therapy). We also compared the determined CAs with the historical controls of healthy volunteers (*N* = 3 mean age: 36.3) (**Supplementary Table S2**).

The mean values of aberrations did not differ for the patient group and the healthy volunteer group. There were 76.0 ± 2.7 vs. 73.7 ± 3.9 total aberrations/100 cells after 3 Gy irradiation and 260.9 ± 7.7 vs. 302.7 ± 13.0 total aberrations/100 cells resulted from 6 Gy *in vitro* radiation (**Supplementary Figure S2**). The patients treated with SBRT in our study were not exceptionally radiosensitive. We also could not identify any outlier radiosensitivity in the treatment group based on CA values after *in vitro* irradiation.

### Chromosomal Aberrations of Patients After Radiotherapy

**Figure 1:**
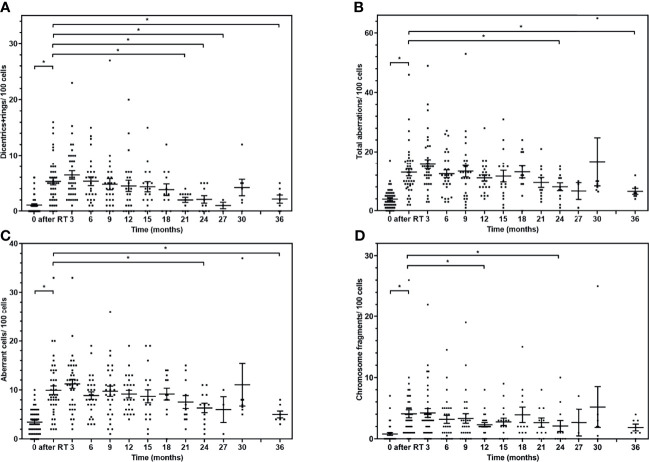
Chromosome aberrations of the lymphocytes of the patients after lung SBRT in follow-up. The individual values and mean (± SE) of chromosome aberrations at different time points. **(A)** Radiation-specific dicentric-plus-ring chromosome frequency is shown at different time points. **(B)** The sum of all aberrations in follow-up. **(C)** The number of cells with any aberration is displayed after radiotherapy. **(D)** The mean frequency of chromosome fragments due to irradiation. The aberration values are compared with the value directly after radiotherapy with Mann-Whitney *U* test (^*^ *p* < 0.05).

We followed the change of chromosomal aberrations in patients after the radiotherapy. The values measured directly after the SBRT can be used to estimate the biological dose, and the samples collected during the follow-up indicate the elimination of radiogenic damage from the blood, which is considered surrogate of the normal tissue toxicities. We observed a 4.8-fold change in the radiospecific dicentric+ring aberrations (from 1.1 ± 0.2 to 5.3 ± 0.6/100 cells, *p* < 0.0001) (**Figure 1A**) and a 3.3-fold total aberration value change (from 3.9 ± 0.5 to 13.0 ± 1.2/100 cells, *p* < 0.0001) after radiotherapy (**Figure 1B**; **Supplementary Table S2**). The first significant decrease (compared with the value directly after radiotherapy) in the aberration values was seen at 21 months after the radiotherapy. There is an increase in CAs at 30 months after radiotherapy, but it is not significant and can be caused by the insufficient number of data points at this time point. In the case of chromosome fragments, the values at 3 years are similar to the ones recorded directly after radiotherapy. The reason for this can be the statistical uncertainty or the damaged lymphocytes—after a period of time—reenter blood circulation from lymph nodes. The CAs did not decrease to the baseline level even 3 years after the radiotherapy.

### Changes of Pulmonary Function Tests After Stereotactic Body Radiotherapy

We compared the mean of the DLCO, FVC, FEV1s, and FEV1% data before and after SBRT therapy (directly and 3, 6, 9, 12, 15, 18, 21, and 24 months after the therapy) and found no significant difference between them.

**Figure 2:**
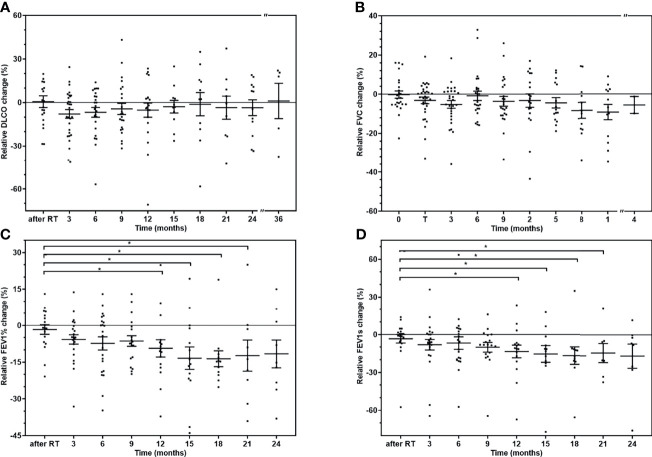
The relative change of the pulmonary function tests of the patients. The individual values and mean (± SD) of pulmonary function tests at different time points. **(A)** Relative DLCO change. **(B)** Relative FVC change. **(C)** Relative FEV1% change. **(D)** Relative FEV1 change. The pulmonary function changes are compared with the value directly after radiotherapy with Mann-Whitney *U* test (^*^ *p* < 0.05).

On the other hand, COPD as a comorbidity can alter baseline pulmonary function data and cause substantial differences between the patients. Therefore, we calculated relative pulmonary function change values: (PFT_time point_ − PFT_baseline_)/PFT_baseline_. There was no significant change between the relative DLCO and FVC alterations directly after radiotherapy and at time points during the follow-up (**Figures 2A, B** **)**. However, there were substantial differences between the patients. The FVC relative increase at 3 months, for example, is between 0% and 10% for the 43.8% and between 10% and 20% for 3.1% of the patients, respectively. At the same time point, there is a 0%–10% decrease for 34.4%; 10%–20% for 12.5%, and 20%–30% for 6.2% of the patients, respectively. However, the average values are negative, showing pulmonary function decrease.

Both the FEV1s and FEV1% relative changes show significant decrease after 12, 15, 18, and 21 months, but there is no difference after 2 years (**Figures 2C, D** **)**. We could not perform any subgroup analysis of the effect of COPD as there were only ten non-COPD patients (20.4%) in our cohort.

### Relationship Between *In Vitro* Chromosomal Aberrations and Pulmonary Function Values of the Patients

**Figure 3:**
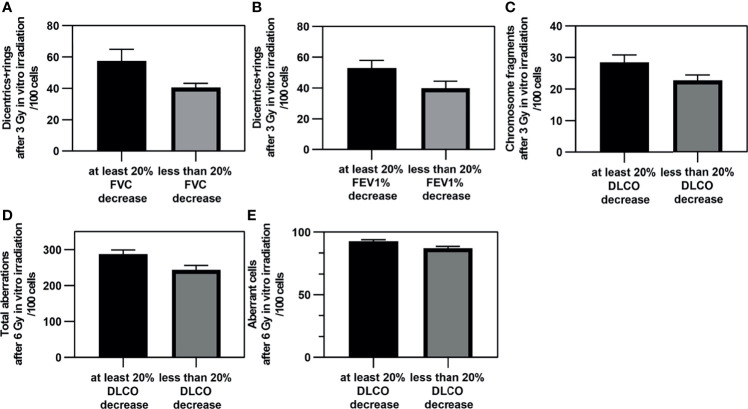
The significant differences in chromosome aberrations between patients with severe and slight pulmonary function decrease. The mean (± SE) of *in vitro* chromosome aberrations of patients before radiotherapy who developed severe or slight/no pulmonary function decrease after radiotherapy of the lung. **(A)** Dicentrics plus rings after 3 Gy and FVC. **(B)** Dicentrics plus rings after 3 Gy and FEV1%. **(C)** Chromosome fragments after 3 Gy and DLCO. **(D)** Total aberrations after 6 Gy and DLCO. **(E)** Aberrant cells after 6 Gy and DLCO. The difference between the chromosomal aberration values are found to be significant with Mann-Whitney *U* test (*p* < 0.05).

We compared the chromosome aberrations measured before radiotherapy in the case of patients who later showed more than 20% of decrease in pulmonary function (at any of the time points) and in the case of those with less decrease (including patients with improving PFTs). There was no significant difference (Mann-Whitney *U* test) in the chromosome aberrations of the patients with at least 20% and of those with less FEV1 decrease. However, we found more dicentrics+rings in the samples irradiated with 3 Gy prior to radiotherapy of patients whose FVC (*p* = 0.010) or FEV1% (*p* = 0.038) later decreased by at least 20% (**Figures 3A, B** **)**. Furthermore, patients with at least 20% decrease in DLCO had significantly more chromosome breaks (*p* = 0.037) after 3 Gy, and total aberrations (*p* = 0.009) and aberrant cells (*p* = 0.012) after 6 Gy *in vitro* irradiation (**Figures 3C–E**).

We found only one case when the dicentrics plus rings frequency was significantly higher in patients with a severe FEV1% decrease than this CA value of healthy donors (*p* = 0.048) (Mann-Whitney two-tailed *t*-test). Chromosme fragment value was also significantly lower in patients with no/slight FEV1 decrease than healthy controls (*p* = 0.049).

**Figure 4:**
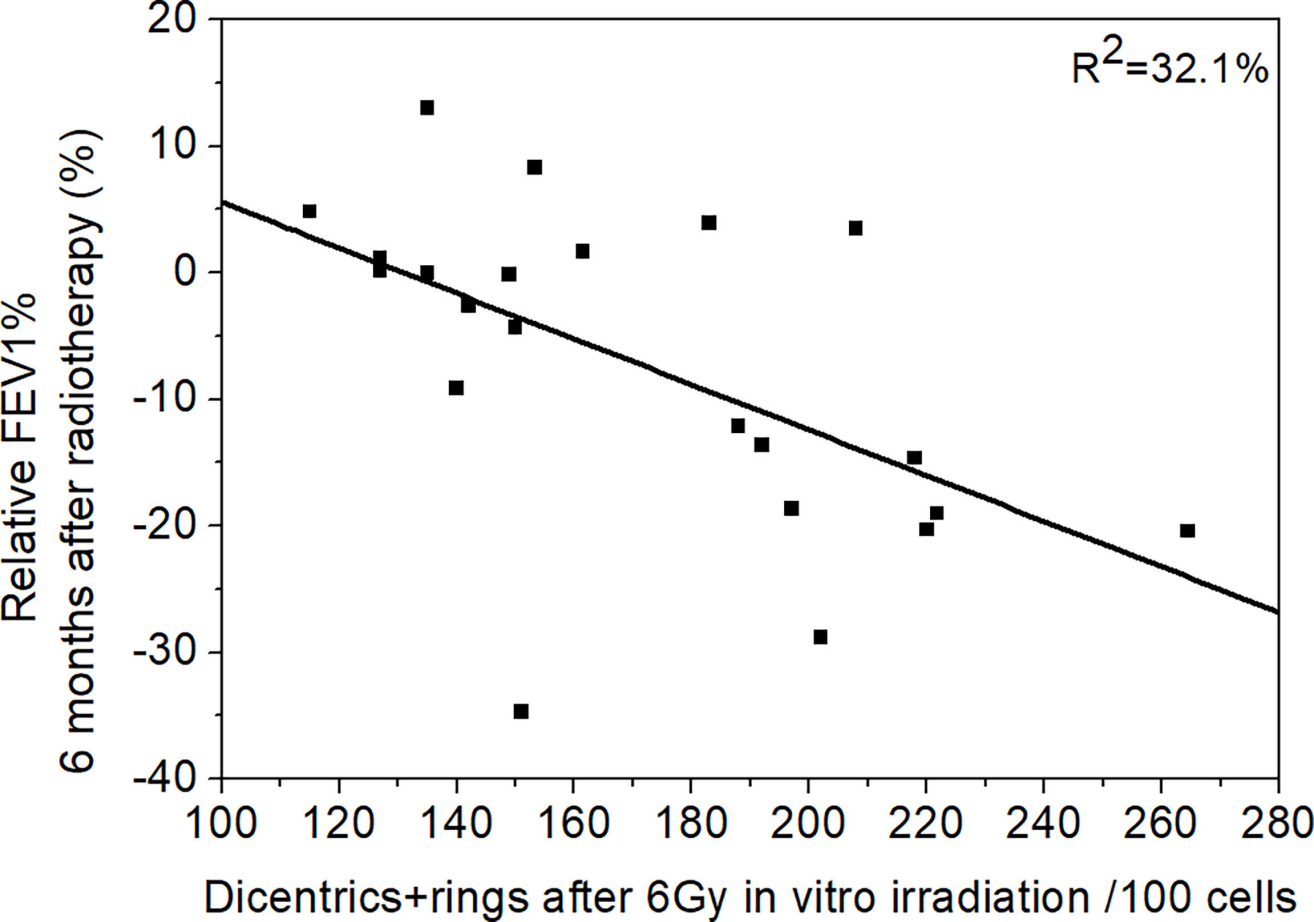
The connection of chromosome aberrations and pulmonary function. The connection between the relative FEV1% 6 months after the radiotherapy and dicentrics and rings after 6 Gy *in vitro* irradiation. The FEV1% decrease is bigger when there are more chromosome aberrations in the *in vitro*-irradiated blood. Regression line was fitted (*R* ^2^ = 32.1%).

We analysed the predictive capacity of chromosome aberrations on pulmonary function by performing univariate regression analysis. There were twelve significant models (**Supplementary Table S3**). All the regression coefficients are negative: the more chromosome aberrations generated (by *in vitro* irradiation) in the blood taken before radiotherapy, the more lung function decrease was seen in the patients (example in **Figure 4**). Approximately 19%–35% (*R* ^2^) of the pulmonary function variance can be explained by the change of the chromosome aberrations of *in vitro*-irradiated blood. The DLCO and FEV1% values were predicted in the majority of the models. Furthermore, chromosome aberrations after 3 Gy irradiation were predictors in as many models as CAs after 6 Gy irradiation.

### Connection Between the *In Vitro* and the *In Vivo* Chromosome Aberrations

**Figure 5:**
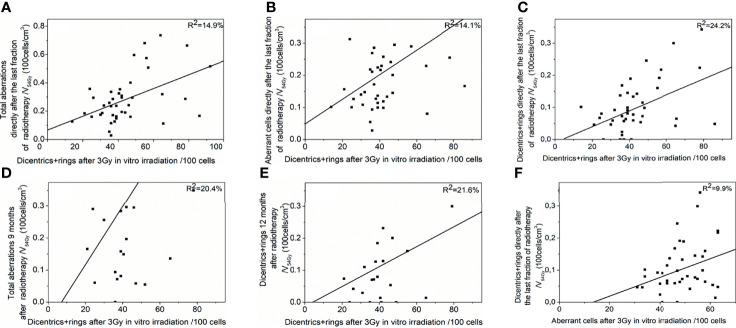
Connection between chromosome aberrations after the *in vitro* and the *in vivo* irradiation. Chromosome aberrations of *in vitro*-irradiated blood taken before radiotherapy could predict chromosome aberrations resulted from lung stereotactic radiotherapy of the patient. Significant regression models are shown: Dicentrics plus rings after 3 Gy predict **(A)** total aberrations, **(B)** aberrant cell frequency, **(C)** dicentrics plus rings directly after the last fraction of radiotherapy **(D)** total aberrations 9 months after therapy **(E)** dicentrics plus rings 12 months after therapy. **(F)** Aberrant cell frequency after 3 Gy can predict dicentrics plus rings directly after the last fraction of radiotherapy.

We compared the chromosome aberration values measured in the *in vitro*-irradiated blood taken from the patients before therapy and the CAs measured from the blood of the patients after radiotherapy. As the CA values are affected by the irradiated volume, we divided the *in vivo* CA data of all time points with the V_54 Gy_ isodose volume (the volume, which obtained at least the 90% of the prescribed dose) of the same patient in order to normalise them. We found six significant models, which suggest that the CA values measured in the *in vitro* and in the *in vivo* blood samples are connected (**Supplementary Table S4**). Dicentrics plus rings after 3 Gy irradiation predicted CAs/V_54 Gy_ measured directly after the radiotherapy: total aberrations (*p* = 0.015, *R* ^2^ = 14.9%), aberrant cell value (*p* = 0.022, *R* ^2^ = 14.1%) and dicentrics plus rings (*p* = 0.001, *R* ^2^ = 24.2%) (**Figures 5A–C**). The connection of dicentrics plus rings after 3 Gy irradiation and total aberrations 9 months later/V_54 Gy_ arose significantly in a regression model (*p* = 0.020, *R* ^2^ = 20.4%) (**Figure 5D**). Furthermore, the same *in vitro* CA predicted dicentrics plus rings 12 months after the radiotherapy/V_54 Gy_ (*p* = 0.034, *R* ^2^ = 21.6%) (**Figure 5E**). There was a significant connection between aberrant cell values after 3 Gy irradiation and dicentrics plus rings directly after the radiotherapy/V_54 Gy_ (*p* = 0.045, *R* ^2^ = 9.9%) (**Figure 5F**) as well.

## Discussion

The treatment of primary lung cancer with SBRT is proved to result in exceptional local progression-free survival (LPFS). The LPFS at 2 years was 64%–96% and 52%–95% at 3 years in large cohorts. A European study demonstrated even more than 90% local control at 3 years for a regimen of 60 Gy in eight fractions (32). The overall survival was between 52% and 87% at 2 years, and between 43% and 75% at 3 years. SBRT increased 3-year overall survival by 14%–30% compared with conventional radiotherapy in NSCLC (7, 33). Our result of overall (68.9% at 36 months) and lung cancer-specific survival (83.6% at 36 months) (**Supplementary Figure S1**) are in line with the meta-analysis of Prezzano et al. who described a 43%–95% overall survival at 3 years and local control even as high as 98% (34).

Meanwhile, severe (≥grade 3) side effects in a wide variety of studies occurred in 0%–3% (mostly CTCAE graded) (35–38). On the other hand, there were controversial results of change in pulmonary function after lung radiotherapy. Ohashi et al., for example, described no change in FEV1 and an increase in DLCO in a small population of Japanese patients (8). However, Henderson et al. observed 1.11 ml/min/mmHg/year DLCO decrease in 70 patients (9). COPD also affected the change of DLCO in the thirty-patient cohort of Bishawi et al. They found an increase of DLCO in patients without COPD (*p* = 0.022), but no change in the subgroup suffering from COPD (39). In another study, FEV1s decreased by 5.8% and DLCO was reduced by 6.3% at 2 years (10). FEV1 and FVC difference were also recorded in patients free of COPD (7.9% and 5.9%, respectively) (40). Comparing individuals, a minimum of 19.4% DLCO decrease found in 10% of the patients and at least 18% increase was found in another 10% of the patients by Stephans et al. (11). We could not find any significant difference between the absolute PFT results before therapy and at any other time points. On the other hand, there was a substantial difference between the patients. We observed even 20%–30% changes in approximately 5% of the patients at certain time points. The occasional increase might be explained by the shrinking of tumours, which previously obstructed airways or vessels.

One of the limitations of our study is this large difference between the PFT patients. Unfortunately, the number of patients was also limited. Therefore, to equilibrate the interpersonal variability of the baseline PFTs, we applied relative change values: baseline subtracted from the PFT value of the time point and normalised with the baseline data. The relative changes of all types of pulmonary function test show decrease after the SBRT (**Figure 2**). However, there is no significant change between the time points except in the FEV1 data after 12 months. There was a tendency of greater loss of pulmonary function after 3 months measured with DLCO, which resolved later. At the same time, there was a tendentious steady decrease in FVC and FEV1 values (**Figure 2**). The decrease of FVC and FEV1 could be attributable to the worsening of the COPD disease or continuing smoking (in 77.7% and 22.2% of the patients, respectively). DLCO value is less affected by obstructive conditions and might indicate the temporary effect of the radiotherapy.

Identification of patients with extreme radiosensitivity with *in vitro* tests would reduce the frequency of severe side effects and healthcare cost as well. The possibility of evaluation of radiosensitivity with biodosimetric methods was previously studied. For instance, the number of lethal defects in the lymphocytes after the irradiation of blood taken from patients prior to radiotherapy may also be related to the risk of fibrosis (19). The frequency of chromosome aberrations in peripheral blood taken from breast cancer patients revealed that patients with higher chromosome aberration frequencies had more severe acute toxicity. There was also a correlation between the number of translocations and the time course of the side effects of the skin (22). It was also suggested that chromosome aberrations should be used to predict acute side effects (21).

In our study, we could not find extreme elevated radiosensitivity, i.e., chromosomal aberrations of the patient group were similar to chromosomal aberrations of healthy volunteers (**Supplementary Figure S2**). We showed multiple connections based on regression models between *in vitro* chromosome aberrations and pulmonary lung function test results (**Figure 4** and **Supplementary Table S3**). Our data suggest that the chromosome aberration method has predictive value in assessing personal radiosensitivity during SBRT lung radiotherapy. To the best of our knowledge, our study is the first in attempting to prove this connection. As the method is cheap and easily adaptable to every laboratory, it can be a competitor to the highly expensive molecular genetic techniques. We detected multiple differences in the chromosome aberrations after *in vitro* irradiation in patients who later developed more than 20% decrease in their pulmonary function test at any time point of the follow-up. Most of the difference was found when we stratified the patients according to DLCO decrease. We also applied regression analysis, and most significant models we built applied the sum of dicentric and ring chromosomes as a predictor. Dicentrics and rings are chromosome aberrations specifically generated as a result of ionising radiation. We observed connections with the early (directly after radiotherapy and 3 months later) and late toxicities as well. We found the most connections at 6 months. The *in vitro* irradiation with 3 Gy was predictive in as many models as the CA values after 6 Gy (**Supplementary Table S3**). Borgman et al. found that higher dose was better for *in vitro* irradiation aiming at personal sensitivity assessment, but they described connections only with acute side effects (21).

We could find significant regression models of *in vitro* chromosomal aberrations predicting *in vivo* CAs (**Figure 5** and **Supplementary Table S4**) in line with former studies (41, 42). To exclude the effect of volume, we normalised the *in vitro* CA data with the irradiated volume of the given patient. We did not use the size of the PTV because the lymphocytes can be damaged by the radiation anywhere in the irradiated field, not just in the PTV. The most frequent connections were again between the radiation-specific dicentrics and rings and other CAs.

We also observed significant differences in the total aberration value between baseline and 24- as well as 36-month time points (**Figure 1**). Seemingly, the aberrations did not clear out from the blood for 3 years. Similar results were published with micronucleus method after rectal cancer radiochemotherapy (42) and miscellaneous tumour entities (43) for 2 years and 19–75 months, respectively.

In conclusion, the lack of decrease of the absolute pulmonary function data shows that in our cohort, lung SBRT was a safe alternative of surgery in comorbid patients. However, in our cohort, the decrease of the relative pulmonary performance could be predicted by chromosome aberrations. Furthermore, we showed that there are more chromosome aberrations in the *in vitro*-irradiated blood (taken before radiotherapy) of the patients who later developed at least 20% relative pulmonary function loss. Our results can be suitable—after further study and validation—for prediction of individual radiosensitivity and pulmonary function decrease caused by chest irradiation.

## Data Availability Statement

The raw data supporting the conclusions of this article will be made available by the authors, without undue reservation.

## Ethics Statement

The studies involving human participants were reviewed and approved by the Hungarian Ethical Committee, ETT TUKEB (23546-3/2017/EKU). The patients/participants provided their written informed consent to participate in this study.

## Author Contributions

ZK, GF, MK-G, and SG performed the laboratory experiments. TP and AH applied the *in vitro* tests. AB, KL, GO, and ZM performed follow-up. ZK, JL, and ZJ wrote the manuscript. ZT-N and CP supervised the work. All authors contributed to the article and approved the submitted version.

## Funding

This study was supported by the Hungarian Thematic Excellence Programme (TKP2020-NKA-26), the National Laboratories Excellence program (under the National Tumorbiology Laboratory project (NLP-17)), and the National Research Development and Innovation Office under Grant [K-124937].

## Conflict of Interest

The authors declare that the research was conducted in the absence of any commercial or financial relationships that could be construed as a potential conflict of interest.

## Publisher’s Note

All claims expressed in this article are solely those of the authors and do not necessarily represent those of their affiliated organizations, or those of the publisher, the editors and the reviewers. Any product that may be evaluated in this article, or claim that may be made by its manufacturer, is not guaranteed or endorsed by the publisher.

## Supplementary Material

Supplementary Figure 1 Survival data after SBRT of the lung cancer patients Survival outcome data of the lung cancer patients of our study after stereotactic radiotherapy: **(A)** Local relapse free survival **(B)** Regional relapse free survival **(C)** Distant metastasis free survival **(D)** Survival without new treatment **(E)** Lung cancer specific survival **(F)** Overall survival.

Supplementary Figure 2 Chromosome aberrations of the lymphocytes after in vitro irradiation in SBRT patients and healthy volunteers. The mean of chromosome frequencies (± SE) after 3 and 6 Gy *in vitro* irradiation of healthy controls and lung cancer patients are shown at different time points.
