# Supplementary figures and images for: Chromosomal Aberrations in Blood Lymphocytes as Predictors of Respiratory Function After Stereotactic Lung Irradiation
Source: Front Oncol. 2022 Jan 27;11:829972. doi: 10.3389/fonc.2021.829972 (PMC8828562; doi:10.3389/fonc.2021.829972)

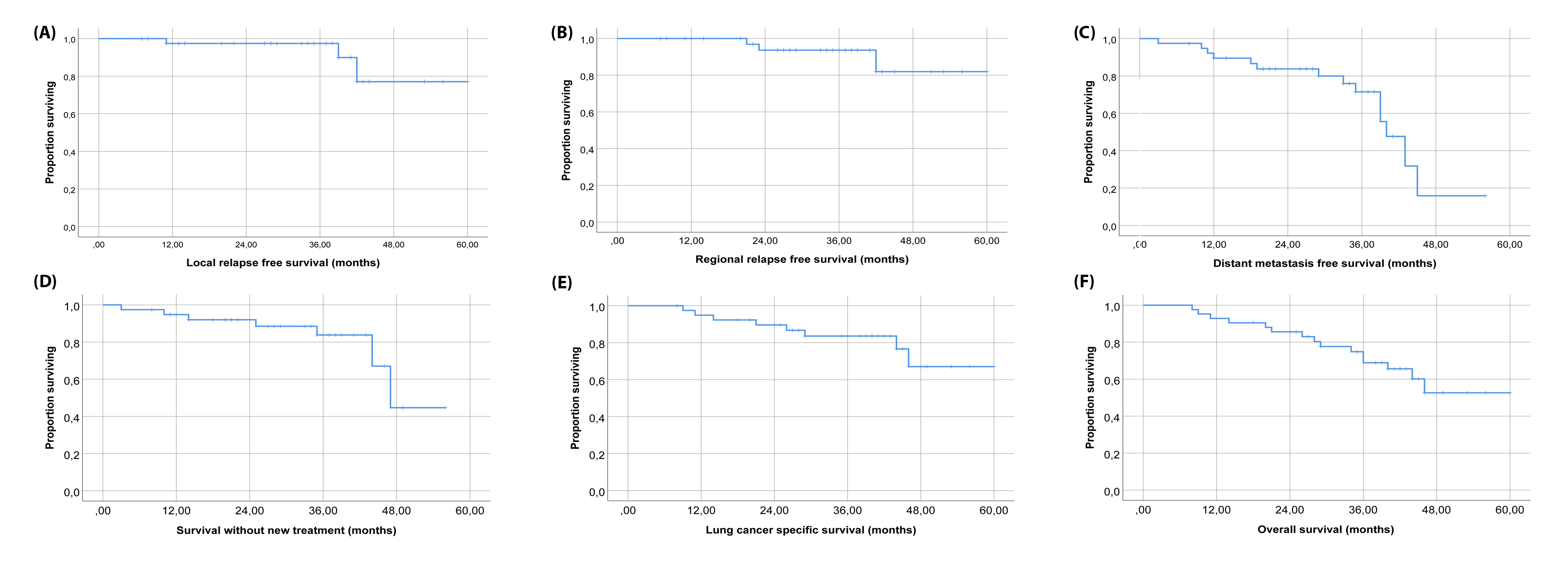

Supplement: Supplementary Figure 1 — Survival data after SBRT of the lung cancer patients Survival outcome data of the lung cancer patients of our study after stereotactic radiotherapy: (A) Local relapse free survival (B) Regional relapse free survival (C) Distant metastasis free survival (D) Survival without new treatment (E) Lung cancer specific survival (F) Overall survival. [file Image_1.tif]
